# Genomic Landscape Underlying Biofilm Formation Diversity in *Nakaseomyces glabratus* Clinical Isolates

**DOI:** 10.1002/mbo3.70444

**Published:** 2026-10-21

**Authors:** Inês Vieira Costa, Maria Zolotareva, Mafalda Cavalheiro, Dalila Mil‐Homens, Isabel M. Miranda, Kaname Sasamoto, Arsénio Fialho, Acácio G. Rodrigues, Hiroji Chibana, Miguel Cacho Teixeira

**Affiliations:** ^1^ Department of Bioengineering, Instituto Superior Técnico Universidade de Lisboa Lisbon Portugal; ^2^ iBB ‐ Institute for Bioengineering and Biosciences, Associate Laboratory i4HB‐Institute for Health and Bioeconomy Instituto Superior Técnico Lisbon Portugal; ^3^ MISE‐Health, Faculty of Medicine University of Porto, Alameda Prof Hernâni Monteiro Porto Portugal; ^4^ Medical Mycology Research Center (MMRC) Chiba University Chiba Japan

## Abstract

Biofilm formation is a key determinant of persistence and pathogenicity in fungal infections, yet the genetic basis underlying its variability among clinical isolates remains poorly understood. In this study, a collection of 95 *N. glabratus* clinical isolates was analyzed in terms of biofilm formation capacity, revealing large phenotypic diversity. Interestingly, urinary tract associated isolates were found to produce, on average, twice as much biofilm as the remaining isolates. A more in‐depth analysis was carried out focusing on a selection of three of the lowest biofilm producing and four of the highest biofilm producing isolates. Higher total biofilm mass was found to consistently correlate with higher metabolic activity and carbohydrate extracellular matrix content, while no correlation with virulence against the *Galleria mellonella* infection model could be detected. Whole‐genome sequencing of these high and low biofilm producing strains led to a list of 184 genes whose mutation may underlie biofilm formation variability. Deletion of 24 of these genes, identified *BST1*, *EST3*, *LSM7*, *AMS1* and *ZCF21* as previously uncharacterized contributors to biofilm formation, whose disruption results in a measurable reduction in biofilm biomass. Altogether, the obtained knowledge suggests within‐host positive selection in the urinary tract, for *N. glabratus* specialization towards biofilm growth. The identification of new effectors of biofilm formation is consistent with the existence of a multi‐layered network that connects transcriptional regulation and cell surface architecture in biofilm development.

## Graphical abstract

*N. glabratus* is the second most common cause of candidiasis and uses adhesion to human epithelia and medical devices to promote persistent infections. By analysing the diversity of biofilm formation capacity among nearly 100 clinical isolates, and addressing the underlying genetic basis, new players in biofilm formation were identified.

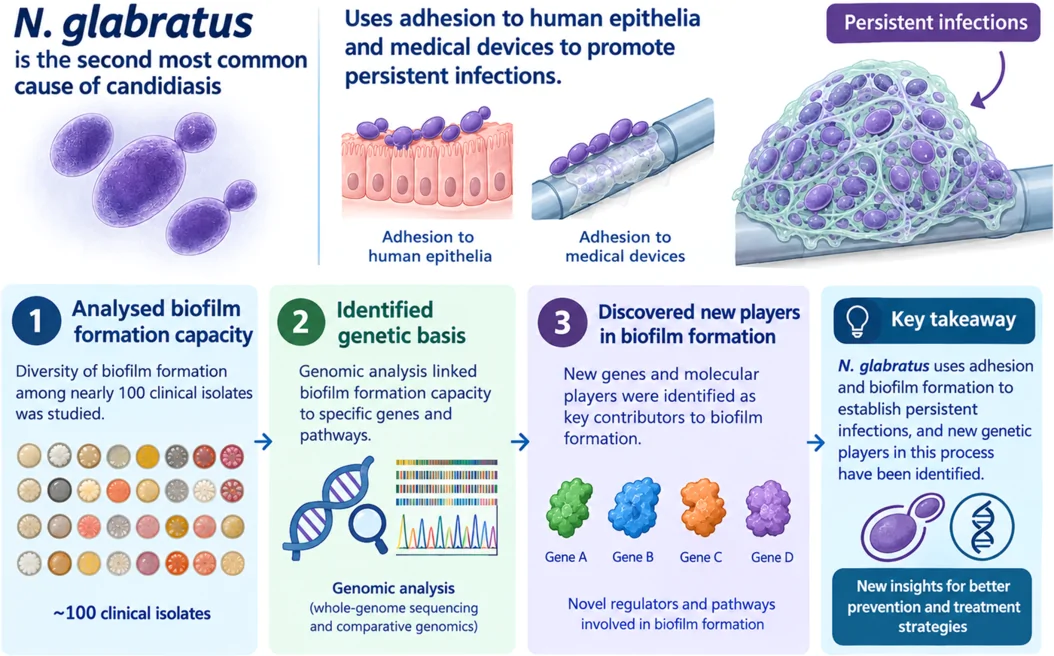

## 1 Introduction

Pathogens belonging to the *Candida* genus are notorious for their ability to form biofilms. The adhesive strength aids in body colonization and infection, while the protective nature of the secreted matrix impermeabilize the cells against the immune system and conventional therapeutics. *Nakaseomyces glabratus* (former *Candida glabrata*) biofilms are of further interest, as the specialization towards stronger biofilms seems to be phylogenetically correlated with the emergence of the pathogenic lifestyle in its clade (Roetzer et al. 2011; Gabaldón et al. 2013). For instance, the main genetic differences observed between *N. glabratus*, and all its environmental, non‐pathogenic closely related species, including *Saccharomyces cerevisiae*, lie in expansions in cell‐wall organization and adhesion gene families, particularly those of the EPA family, mostly encoded in the highly dynamic sub‐telomeric regions.

These observations resulted from comparing the reference genomes of related species with contrasting ecological strategies. Moreover, recent advances in sequencing technologies enabled the inclusion of a greater number of representative *N. glabratus* clinical strains to the studies. Those contributed to understanding virulence (Healey et al. 2016; Carreté et al. 2018), drug resistance (Pais et al. 2022; Galocha et al. 2022), and even within‐host microevolution in this species (Carreté et al. 2019b; Helmstetter et al. 2022). As it appears, in‐host evolution manifests itself mostly through enrichment of non‐synonymous mutations in genes encoding cell‐wall proteins and adhesins (Carreté et al. 2019b; Helmstetter et al. 2022), drawing attention, once again, towards the rather intriguing adhesion and biofilm phenotypes.

Ecological drivers behind in‐host biofilm formation had been raising questions for a long time. In 2002, Shin et al observed that some *Candida* strains isolated from bloodstream produced more biofilm than those isolated from other sites (Shin et al. 2002). In their analysis, this was verified for *C. tropicalis* and *C. parapsilosis*, but not for *N. glabratus*. In 2022, Domingues et al evaluated whether *Candida* species isolated from the oral mucosa of diabetic patients would have increased biofilm formation capacity (Domingues et al. 2022). The hypothesized correlation was verified in *C. albicans* strains, but once again, not in *N. glabratus*. Interestingly, in 2005, the work of Domergue et al. established a regulatory link between biofilm initiation and pathogen's installation in the urinary tract of mammals, through NAD dependent telomeric silencing (Domergue et al. 2005). However, no evidence for an ecological correlation between this environment and biofilm formation variability in *N. glabratus* had been obtained.

Formation of biofilms in *N. glabratus* is often associated with adhesins. This species encodes for several adhesins in its genome, belonging to three adhesin families, but the most studied are the EPA (epithelial adhesin) gene family, particularly *EPA1*, *EPA6*, and *EPA7*, which promote adherence to abiotic surfaces and host tissues While Epa6 plays a major role in biofilm development, Epa1 is associated with epithelial adhesion, contributing substantially to overall adherence capacity (Ielasi et al. 2012; Wang et al. 2024). These adhesins are located in sub‐telomeric regions of the genome and are tightly regulated by chromatin‐based silencing mechanisms. Biofilm formation has been shown to rely on transcriptional remodeling occurring under the control of the Sir‐dependent silencing complex, which governs *EPA* gene expression and enables rapid adaptation through modulation of sub‐telomeric loci (Rosas‐Hernández et al. 2008; Riera et al. 2012; Cavalheiro and Teixeira 2018). This regulatory framework is further complemented by transcription factors such as Efg1 and Tec1, which, although best characterized in other *Candida* species, are part of conserved regulatory circuits controlling adhesion, extracellular matrix (ECM) production, and biofilm architecture (Finkel and Mitchell 2011; Barros et al. 2016; Cavalheiro et al. 2021). Together, these pathways enable *N. glabratus* to establish structurally robust and drug‐tolerant biofilm communities.

In this study, a library of 95 clinical *N. glabratus* isolates from patients attending two major Hospitals in Portugal, were screened for biofilm formation capacity. Correlation between biofilm formation and azole drug susceptibility, host's age and sex, and colonization/infection site was evaluated. Whole‐genome sequencing of representative high and low biofilm producing strains and comparative genomic scan, centered on point mutation annotation and effect prediction, was carried out to identify genes whose mutation may underlie the observed biofilm formation variability. The impact of the deletion of several of these putative biofilm formation‐related genes was scrutinized.

## 2 Materials and Methods

### 2.1 Strains and Growth Medium

A collection of 95 *N. glabratus* clinical isolates, collected from patients admitted to Hospital of Santa Maria (HSM), Lisboa, and Centro Hospitalar São João (CHSJ), Porto, was used in this study (Costa‐de‐Oliveira et al. 2008; Faria‐Ramos et al. 2014; Costa et al. 2016). This collection was previously characterized in terms of resistance to antifungal drugs, namely fluconazole, and clotrimazole (Costa et al. 2016) as summarized in Table S1.

Cells were batch‐cultured in the following media: Yeast extract Peptone Dextrose (YPD), containing 20 g glucose, 20 g peptone and 10 g of yeast extract per liter of deionized water; or Sabouraud Dextrose Broth (SDB), containing 40 g glucose and 10 g of meat peptone, pH adjusted to 5.6. Cells were stored in YPD with glycerol 46%, at −80°*C*. To thaw, cells were plated in YPD agar plates and incubated for 24 h at 30°C.

For the adhesion assays, the VK2/E6E7 human epithelium cell line (ATCC CRL‐2616) was used. Cells were maintained with KSF medium, supplemented with 0.1 ng/mL human recombinant epidermal growth factor, 0.05 mg/mL bovine pituitary extract, and 441.1 mg/L calcium chloride. Cells were incubated at 37°C with 5% CO_2_.

### 2.2 Deletion Mutant Construction

Deletion of *SIP3* (*CAGL0J07942g*), *ACK1* (*CAGL0J09702g*), *MFG1* (*CAGL0JK04873g*), *SIR4* (*CAGL0K11396g*), *CAGL0G07645g*, *RER1* (*CAGL0C01837g*), *TAF6B* (*CAGL0F07381g*), *KSP1* (*CAGL0F03311g*), *CAGL0K07502g*, *BST1* (*CAGL0K12408g*), *SWF1* (*CAGL0B01991g*), *EST3* (*CAGL0C03828g*), *SRB2* (*CAGL0D04136g*), *ADA3* (*CAGL0E00693g*), *RPL22B* (*CAGL0F01683g*), *DYN2* (*CAGL0F03091g*), *SKN7* (*CAGL0F09097g*), *LSM7* (*CAGL0J05764g*), *SET1* (*CAGL0L12980g*), *CRZ1* (*CAGL0M06831g*), *YAP6* (*CAGL0M08800g*), *NHP10* (*CAGL0M10505g*), *AMS1* (*CAGL0E05148g*) and *ZFC21* (*CAGL0H01683g*) was carried out by homologous recombination with appropriate DNA cassettes, amplified by polymerase chain reaction (PCR) using the pHIS906 plasmid containing HIS3, in laboratory strain KUE‐100, as described previously (Ueno et al. 2007). The PCR primers used to generate the replacement cassette for each gene and the primers used for PCR confirmation of gene deletion are present in Table S2.

### 2.3 Biofilm Quantification

Biofilm formation in polystyrene microtiter plates was quantified using the crystal violet biofilm assay. The crystal violet solution contained 1% crystal violet (10 g dissolved in 200 mL 96% ethanol) and 0,8% ammonium oxalate monohydrate (8 g dissolved in 800 mL of deionized water).

*N. glabratus* clinical isolates and strains were grown in SDB medium and collected in mid‐exponential phase by centrifugation. Cells were then inoculated in 96‐well polystyrene plates, in 200 µL of SDB medium, with an initial OD_600_ = 0.05 ± 0.005, at 30°C for 24 h, without agitation. After incubation, each well was washed three times with deionized water, to remove cells unattached to the biofilm. Then, the biofilms were exposed to 100 µL of the crystal violet solution for 15 min at room temperature. Afterwards, the solution was discarded, and the biofilms washed another three times with water, to remove any dye not bound to the biofilm. Plates were air dried, and 200 µL of ethanol 70% was added to solubilize the dye, for 10 min. Afterwards, the ethanol and crystal violet were resuspended and homogenized before measuring the absorbance at 590 _nm_ in a microplate reader.

### 2.4 Growth Curves

The relative fitness of wild‐type KUE‐100, and derived *∆ams1*, *∆est3*, *∆lsm7*, *∆bst1*, *∆zcf21* single mutant strains, was evaluated through batch‐culture, in SDB medium, at 30°C, with orbital agitation (250 rpm). The cell suspensions used to inoculate SDB liquid medium were prepared by cell cultivation in SDB medium until standardized culture OD_600nm_ of 0.2 ± 0.02 was reached. Growth curves were followed by measuring culture OD_600nm_.

### 2.5 Cell Viability Assay

Cell viability within the biofilm, was assessed using the Presto Blue Cell Viability Assay. *N. glabratus* clinical isolates and strains were grown in SDB medium and collected in mid‐exponential phase by centrifugation. Cells were then inoculated in 96‐well polystyrene plates, in 200 µL of SDB medium, with an initial OD_600_ = 0.05 ± 0.005, at 30°C for 24 h, without agitation. After incubation, each well was washed three times with sterile phosphate‐buffer saline (PBS) (8 g NaCl, 0,2 g KCl 1.81 g NaH_2_PO_4_.H_2_O, 0.24 g KH_2_PO_4_ per liter of deionized water), to remove cells unattached to the biofilm. Then, the biofilms were incubated in 100 µL of 1:10 Presto Blue Reagent and SDB medium solution, at 30°C for 30 min. After resuspension of the solution, the absorbance at 570 and 600 nm was measured in a microplate reader.

### 2.6 Epithelial Cell Adhesion Assays

To test the ability of select *N. glabratus* clinical isolates to adhere to epithelial cells, VK2/E6E7 human vaginal epithelium cells were grown in 24‐well plates with a 2.5 × 10^5^ cell/mL density. Simultaneously, select *N. glabratus* clinical isolates were grown in YPD medium and collected in mid‐exponential phase by centrifugation on the following day. The mammalian cell culture medium was discarded, and fresh medium was added to each well. Then the *N. glabratus* isolates were added with a density of 12.5 × 10^5^ CFU/well, which corresponds to a MOI of 10. The plates were incubated at 37°C, 5% CO_2_, for 30 min. Each well was then washed three times with a PBS solution, and then added 500 µL of Triton X‐100 0.5%, followed by incubation at room temperature for 15 min. The resulting cell suspension was recovered, diluted and plated onto YPD agar plates to determine CFU count.

### 2.7 Virulence Assays

To test the virulence of select *N. glabratus* clinical isolates, *Galleria mellonella* larvae was used as an infection model. The larvae were reared on pollen grain and bee wax diet at 25°C without light exposure. Larvae in the last instance stage, weighing 200 ± 25 mg were selected for this assay, and the larvae infection protocol was based on previous work by Dalila Mil‐Homens (Mil‐Homens and Fialho 2012). Select *N. glabratus* clinical isolates were grown in YPD and collected by centrifugation when mid‐exponential phase was reached. Yeast cells were washed and resuspended in PBS, with a final injection load of 5 × 10^6^ cells per injection volume. The larvae were infected with 5 µL of PBS resuspended yeast cells, injected via the hindmost left proleg, previously sterilized with 70% ethanol. Control larvae were injected with sterile PBS in the same manner. The larvae were kept in the dark for 72 h at 37°C, and lethality was assessed in 24 h intervals. Larvae were considered dead when unresponsive to touch.

### 2.8 Extracellular Matrix Characterization

Polysaccharide and protein fractions of the biofilms of select clinical isolates were quantified using the phenol‐sulfuric acid method and Bradford's protein assay, respectively.

*N. glabratus* select isolates were grown in YPD medium and collected in mid‐exponential phase by centrifugation. Cells were then inoculated in 24‐well polystyrene plates, in 1 mL of YPD medium, with an initial OD_600_ = 0.05 ± 0.005, at 30°C for 48 h without agitation, with a washing step at 24 h. After 48 h the medium was discarded, and the biofilm scrapped under 2 mL of a sterile solution of 0.89% NaCl. Following biofilm resuspension, mechanical disruption of the ECM was achieved by 1 min of vortexing at maximum speed, and then centrifugation at 5500 g, for 10 min at 4°C, to separate cells from the matrix components. For each sample, 1 mL of the supernatant was added to 2.5 mL of 95% ethanol and left to precipitate the polysaccharides for 18 h at −20°*C*. The pellets and supernatant were separated, where the supernatant was divided in two fractions of 500 µL, and stored separately, to quantify water‐soluble and membrane proteins. The pellet was resuspended in 1 mL of sterile deionized water. Total polysaccharide quantification was carried out through the phenol‐sulfuric acid method. Briefly, 200 µL of 5% phenol and 1 mL of sulfuric acid were added to a glass tube with 200 µL of each sample. The resulting reaction was carried out for 20 min and measured in a spectrophotometer at 490 nm. Glucose was used for the standard curve (0, 10, 50, 100 and 500 µg/mL).

Using the Bradfords protein assay, protein quantification was carried out in 96 well‐plates. Briefly, 40 µL of Bradford reagent were mixed with 160 µL of each sample. Soluble proteins were measured directly from the supernatant sample, while the membrane proteins were previously retrieved from the solid phase, and denatured at 100°C for 1 h. After 15 min of exposure to the reagent, the absorbance of the samples was measured at 595 nm in a spectrophotometer.

### 2.9 Whole‐Genome Sequencing and Data Analysis

Select *N. glabratus* clinical isolates were grown overnight in liquid YPD medium, at 30°C with orbital agitation (250 rpm.). Genomic DNA (gDNA) was extracted using the NZY microbial gDNA isolation kit (NZYTech), following the manufacturer's instructions. Genomic DNA was sequenced on an Illumina HiSeq X platform, producing 2150 base pairs (bp) paired end reads. Library preparation (Nextera XT) and sequencing were carried out by CD Genomics.

Low‐quality bases and adapters were removed using Trimmomatic (v0.38). Read duplicates were removed using PRINSEQ (v0.20.4). The draft genomes were assembled into scaffolds using SPAdes (v3.12.0). Assembly quality was analyzed using QUAST (v.4.6.3). Single nucleotide polymorphisms (SNPs) were identified against the reference genome using BWA (v.0.7.17). and the GATK‐HC (v4.0.8.1). Low‐quality variants were filtered out using BCF tools (v1.9). Finally, variants like SNPs and InDels were identified and annotated using snpEff (v.4.3), using the reference genome for *N. glabratus* (CBS138). snpEff (v.4.3) enabled the prediction of functional consequences of the identified SNPs, through the prediction of non‐synonymous mutations, early STOP codons or frameshifts. Candidate genes were further evaluated based on available gene annotations, predicted functions, and the functions of orthologous genes in *S. cerevisiae*, using information retrieved from the *Candida* Genome Database (Lew‐Smith et al. 2025) and the Saccharomyces Genome Database (Engel et al. 2025).

Raw RNA‐sequencing data is available in NCBI through PRJNA738673 and PRJNA1446863.

## 3 Results

### 3.1 *N. glabratus* Clinical Isolates Harbor Great Biofilm Formation Diversity

A collection of 95 *N. glabratus* clinical isolates was tested for biofilm formation levels, to evaluate the potential phenotypic diversity across strains. These isolates were retrieved from a large pool of body niche samples, including blood, pus, bronchial lavage, sputum, vaginal smear, other mucosal surfaces, urine and feces. 62.5% of sample donors were female, and the age range went from 16 to 93 years, with ~ 50% of the donors being 70+ years old. Crystal violet assays were used in this screening and revealed great diversity in terms of biofilm production capacity, as seen in Figure 1. Most isolates (53%) produce very low levels of biofilm, close to the method's detection limit. Some isolates, however, were found to generate large biofilms, 3% near the method's saturation level.

**Figure 1:**
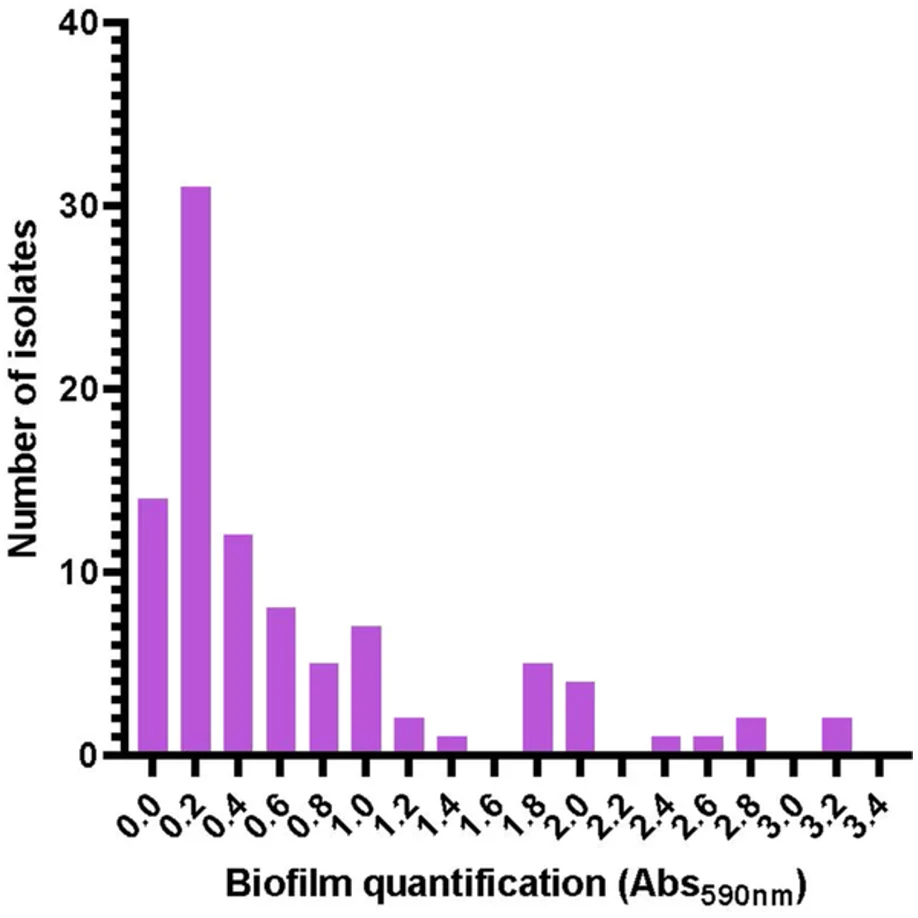
– Histogram of average biofilm quantification among a collection of 95 *N. glabratus* clinical isolates, based on absorbance values at 590 nm (Abs_590nm_), upon the use of the crystal violet assay.

Next, the possible correlation between biofilm formation capacity and the clinical isolate origin was evaluated. In this collection of isolates, biofilm formation capacity does not correlate with age or sex, neither with MIC50 (minimum inhibitory concentration at which 50% of the isolates in a test population are inhibited) values for azole drugs (Table S1). Interestingly, however, urinary tract associated isolates exhibited on average twice as much biofilm total mass, when compared to isolates obtained from other anatomical sites. Indeed, 7 out of the 10 most productive isolates were retrieved from urine samples (Figure 2). Urine biofilms harbored the highest values for mean (0.939) and variance (0.869). The average value of biofilm measured from urine isolates was 1.72 times greater than the mean value for all other samples. These results were found significant, with a *p* = 0.016 performing one‐tailed Welch *T*‐test.

**Figure 2:**
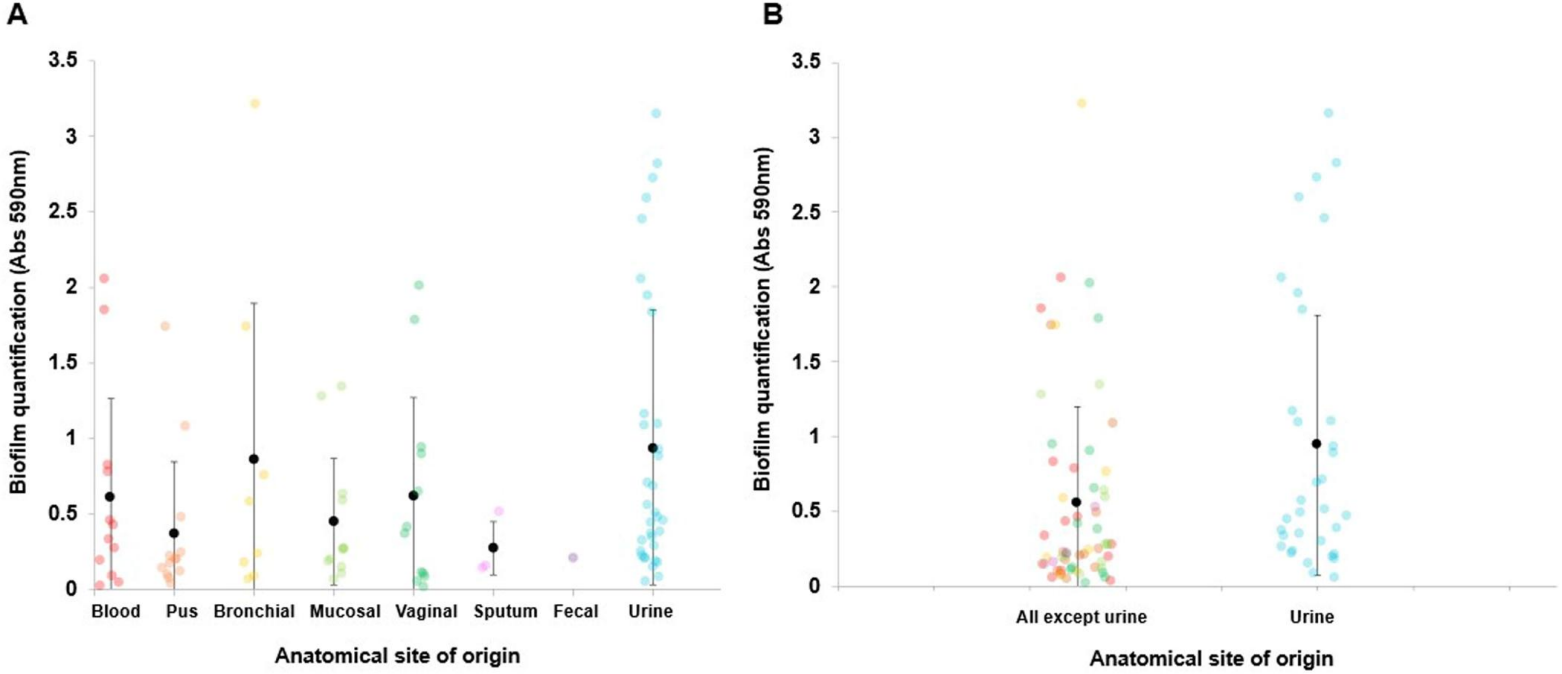
Biofilm quantification, based on absorbance values at 590nm (Abs_590nm_), upon the use of the crystal violet assay, of 95 *N. glabratus* clinical isolates, distributed by anatomical site of origin (A). Average biofilm produced by each strain is represented by a colored dot, with the following color code: red (blood), orange (pus), yellow (bronchial), light green (mucosal), dark green (vaginal), pink (sputum), purple (fecal), blue (urine), black (mean values). Statistical significance (*p* < 0.05) is found comparing biofilms formed by urine derived isolates (average Abs_590nm_ = 0.940) and biofilms derived from all non‐urine clinical samples (average Abs_590nm_ = 0.547) (B).

### 3.2 Higher Biofilm Production Correlates With Higher Polysaccharide Content in the ECM

From the clinical isolate collection, a few strains on both ends of the biofilm producing spectrum (Figure 3), were selected for further comparative studies. Four high biofilm producing isolates (21,461, 37,398, 56,067 and 77,770) and three low biofilm producing isolates (50,570, 67,367 and 73,281) were selected and a series of assays were performed to compare the two groups.

**Figure 3:**
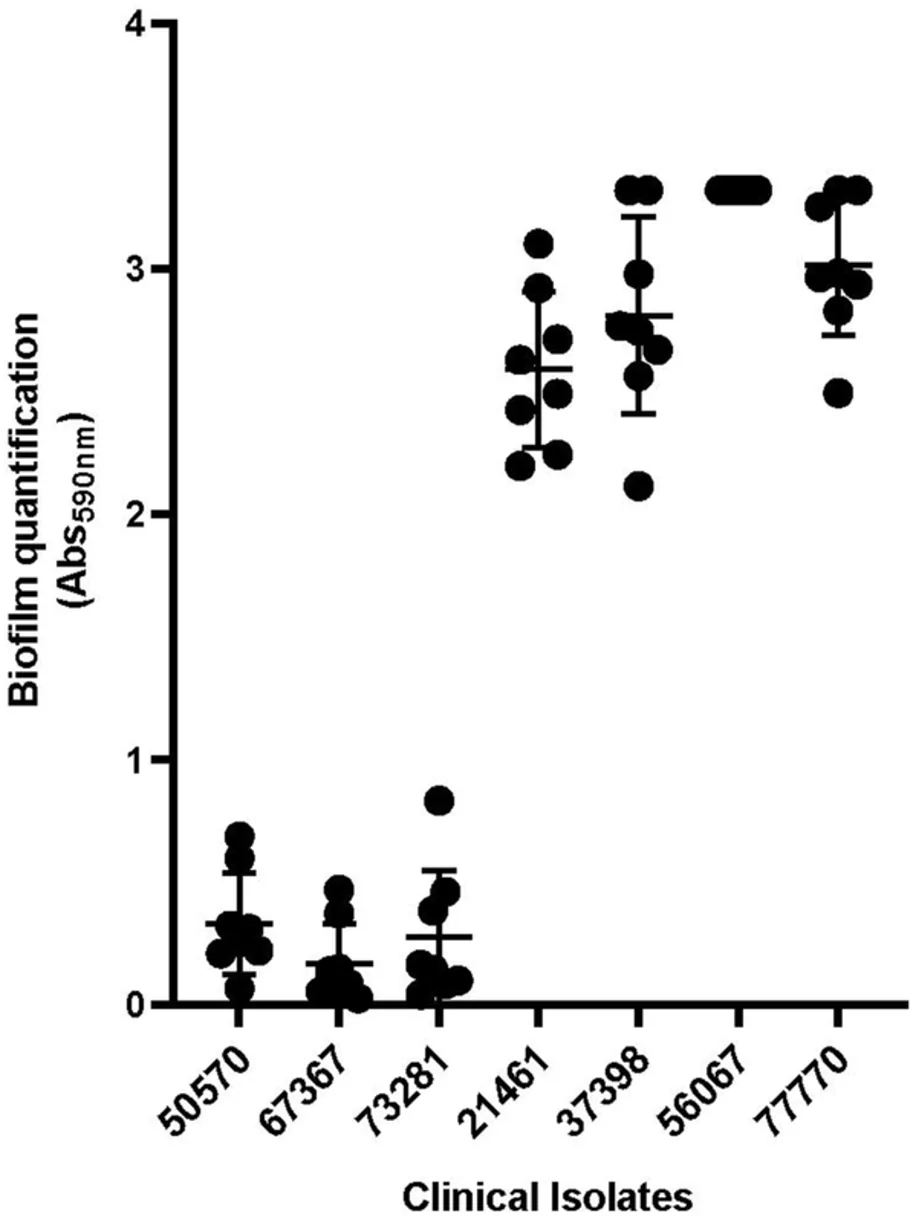
Biofilm quantification, based on absorbance values at 590nm (Abs_590nm_), upon the use of the crystal violet assay, of *N. glabratus* clinical isolates, selected for low (50,570, 67,367 and 73,281) or high (21,461, 37,398, 56,067 and 77,770) biofilm production levels. Dots represent independent replicate quantification, with average values and standard deviations being represented for each strain.

First, the ECM of these isolates was characterized in terms of polysaccharide, soluble and insoluble protein content, as well as cell viability (Figure 4). The matrix of *C. albicans* biofilms is mainly composed of proteins, which account for approximately 55% of its dry weight, exceeding carbohydrate content (Zarnowski et al. 2014). In contrast, the extracellular matrix of *N. glabratus* has been described to have equal amounts of carbohydrate and protein. In the present study this was confirmed only for the weaker biofilm producing strains. In strong biofilm producers, the average of carbohydrates was 5.9 times higher than the concentration of protein. A trend toward lower soluble protein content was observed in the biofilm matrix of the strong biofilm producing isolates 21,461, 37,398, 56,067, 77,770, although this difference did not reach overall statistical significance, due to strain‐to‐strain variability.

**Figure 4:**
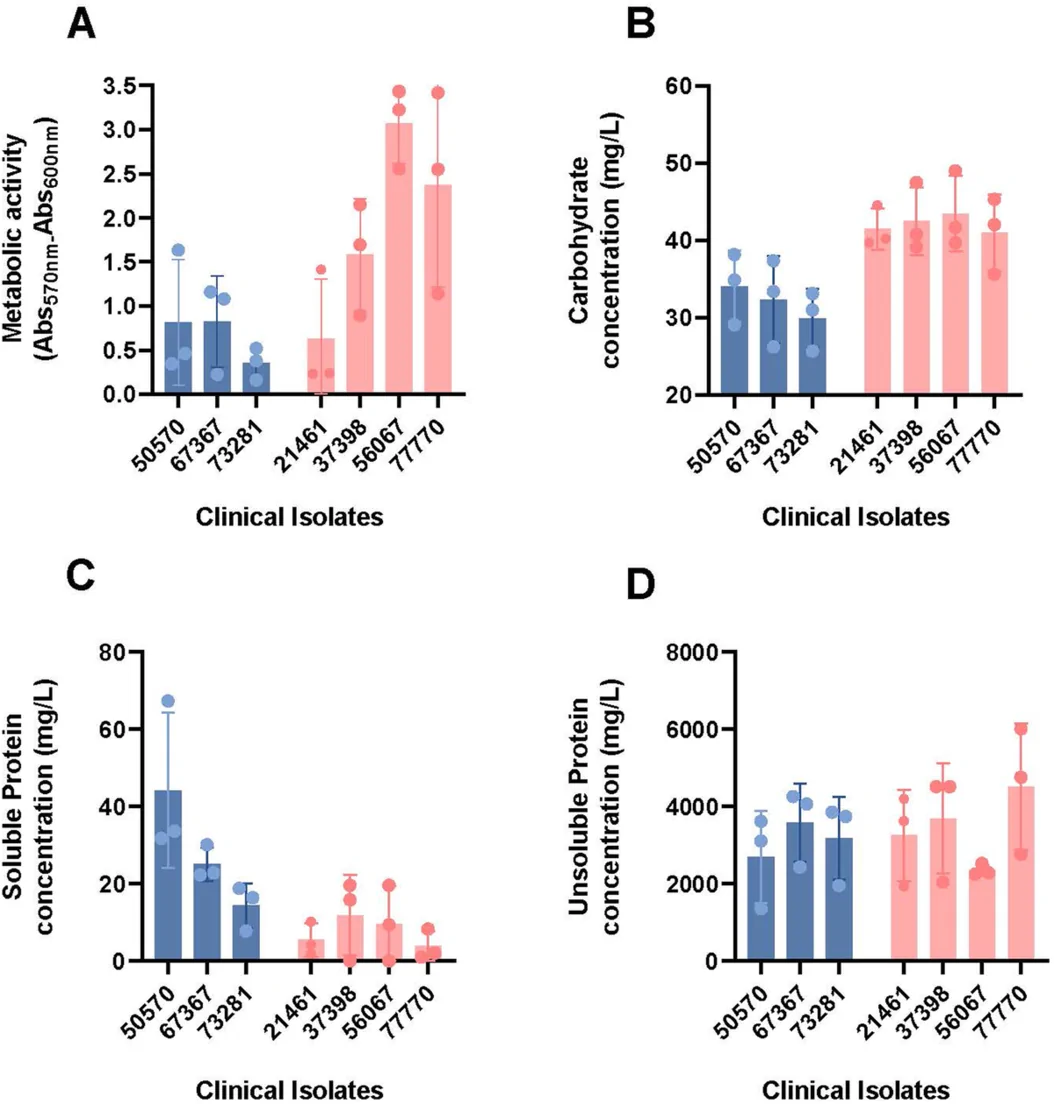
Biofilm characterization, in terms of metabolic activity (A), and total carbohydrate (B), soluble protein (C) and insoluble protein (D) Extracellular Matrix (ECM) concentration, for N. glabratus clinical isolates, selected for their low (50,570, 67,367 and 73,281 – blue bars) or high (21,461, 37,398, 56,067 and 77,770 – pink bars) biofilm production levels. Each circle represents the mean value of one independent biological sample replicate experiment. The bars represent averages of, at least, *n* = 3 independent experiments. Error bars represent the corresponding standard deviations.

Carbohydrate concentrations were consistently higher in the matrices of strong biofilm producers compared with weak biofilm‐producing strains. The average carbohydrate concentration measured in biofilms formed by the strong clinical isolates (21,461, 37,398, 56,067, and 77,770) was 42.1 mg/L, whereas weak biofilm producers (50,570, 67,367, and 73,281) exhibited an average concentration of 32.1 mg/L. These differences were statistically significant (*p* < 0.001), as determined by a one‐tailed Welch's *t*‐test. The average metabolic activity of strong biofilm producers was 2.9‐fold higher than that of weak biofilm producers (*p* < 0.005, one‐tailed Welch's t‐test). All biofilms produced by weak biofilm producing isolates displayed low metabolic activity.

No statistically significant correlation between biofilm formation capacity and cell viability, or soluble or insoluble protein content, could be found. Overall, these results indicate that the strong biofilm phenotype in *N. glabratus* is primarily driven by increased number of biofilm associated cells and synthesis and secretion of carbohydrates, rather than by changes in protein content.

### 3.3 Biofilm Production Capacity Does Not Correlate With Virulence or Adherence to Human Epithelial Cells

The *G. mellonella* infection model was used to evaluate the lethality of *N. glabratus* isolates exhibiting varying biofilm‐forming capacities. Among the clinical isolates tested, a wide range of lethality was observed, but no correlation between biofilm production capacity and infection‐associated mortality could be established (Figure 5).

**Figure 5:**
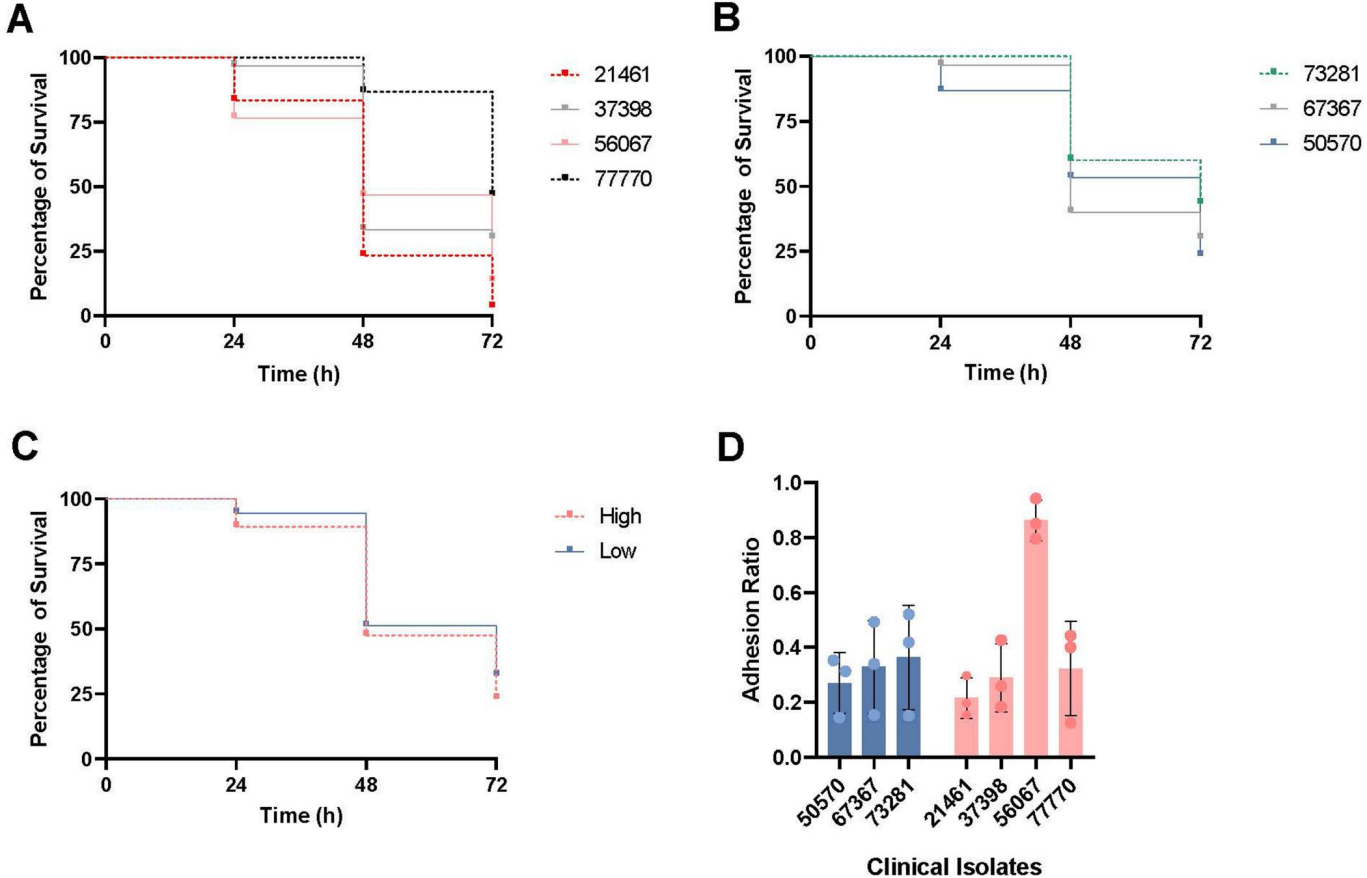
Survival of *G. mellonella* larvae injected with ~5 × 10^7^ CFU/larvae of each indicated high biofilm (A) or low biofilm (B) producing N. glabratus clinical isolate, displayed as Kaplan‐Meier survival curves. (C) Average survival of *G. mellonella* larvae injected with ~5 × 10^7^ CFU/larvae of high biofilm (High) or low biofilm (Low) producing *N. glabratus* clinical isolates, displayed as Kaplan‐Meier survival curves. (D) Adhesion ratio of each indicated high or low biofilm producing *N. glabratus* clinical isolates to VK2/E6E7 human vaginal epithelial cells. The displayed results are the average of at least three independent experiments.

Within the group of strong biofilm producers, lethality ranged from 53% to 97% at 72 h post‐infection, indicating marked strain‐dependent variability. Similarly, among weak biofilm‐producing strains, lethality varied from 60% to 85%. On average, strong and weak biofilm producers caused 77% and 70% mortality, respectively. Survival analysis using the log‐rank (Mantel–Cox) test confirmed that there was no significant difference in infection risk between strong and weak biofilm‐producing *N. glabratus* strains.

Similarly, when assessing the ability of these isolates to adhere to VK2/E6E7 human vaginal epithelial cells, no correlation was observed between increased biofilm production and enhanced adherence. The VK2/E6E7 cell line was used to evaluate the adhesion capacity of the different *N. glabratus* strains to the vaginal epithelium, an important natural colonization site for this species and a reservoir for infection if appropriate conditions arise. Most isolates from both groups exhibited adherence levels ranging between 20% and 50%. Interestingly, the strong biofilm producing isolate 56,067 demonstrated higher adherence values, exceeding 80%. However, this feature was not conserved for the remaining high biofilm producing strains tested herein (Figure 5).

### 3.4 Genomic Diversity Underlying Biofilm Formation Capacity

To assess the genomic variation among clinical isolates exhibiting differential biofilm formation capacity, the genome sequences of the four high biofilm producing strains, 21,461, 37,398, 56,067 and 77,770, and 3 low biofilm producing strains, 50,570, 67,367 and 73,281, were experimentally obtained through Whole‐genome sequencing, and compared with the CBS138 reference *N. glabratus* strain. The objective of this comparison was initially to find SNPs which were present in all high biofilm producing strains and simultaneously absent from the low biofilm producing strains.

When compared to the reference strain, the four high biofilm producing strains share 2660 common SNPs, of which only 10% represented non‐synonymous mutations in transcribed regions. Only two non‐synonymous SNPs were found to be exclusive to all four high biofilm producing strains, when compared to the low biofilm producing isolates: one missense mutation (p.Ala118Val) in ORF *CAGL0L00157g*, encoding a putative adhesin; and missense mutation (p.Phe40Ser) in ORF *CAGL0K07502g*, predicted to encode a protein of unknown function. This approach was found to be too stringent, and further strategies were conceived aiming to relax the selection criteria and admit more genes to the group of possible genes of interest (schematized in Figure 6). Genes with non‐synonymous SNPs, even if not the same SNPs, present in all the high biofilm producers, excluding any genes also found with SNPs in the low biofilm producers where considered, as approach A. This approach led to the identification of 38 genes, whose orthologs and predicted or verified functions are described in Table 1. Another approach (approach B) considered SNPs present in at least 3 of the high biofilm producing isolates, excluding any SNPs also present in the low biofilm producers. This led to 146 genes with mutations with a possible role in the observed biofilm phenotype (Table S3).

**Figure 6:**
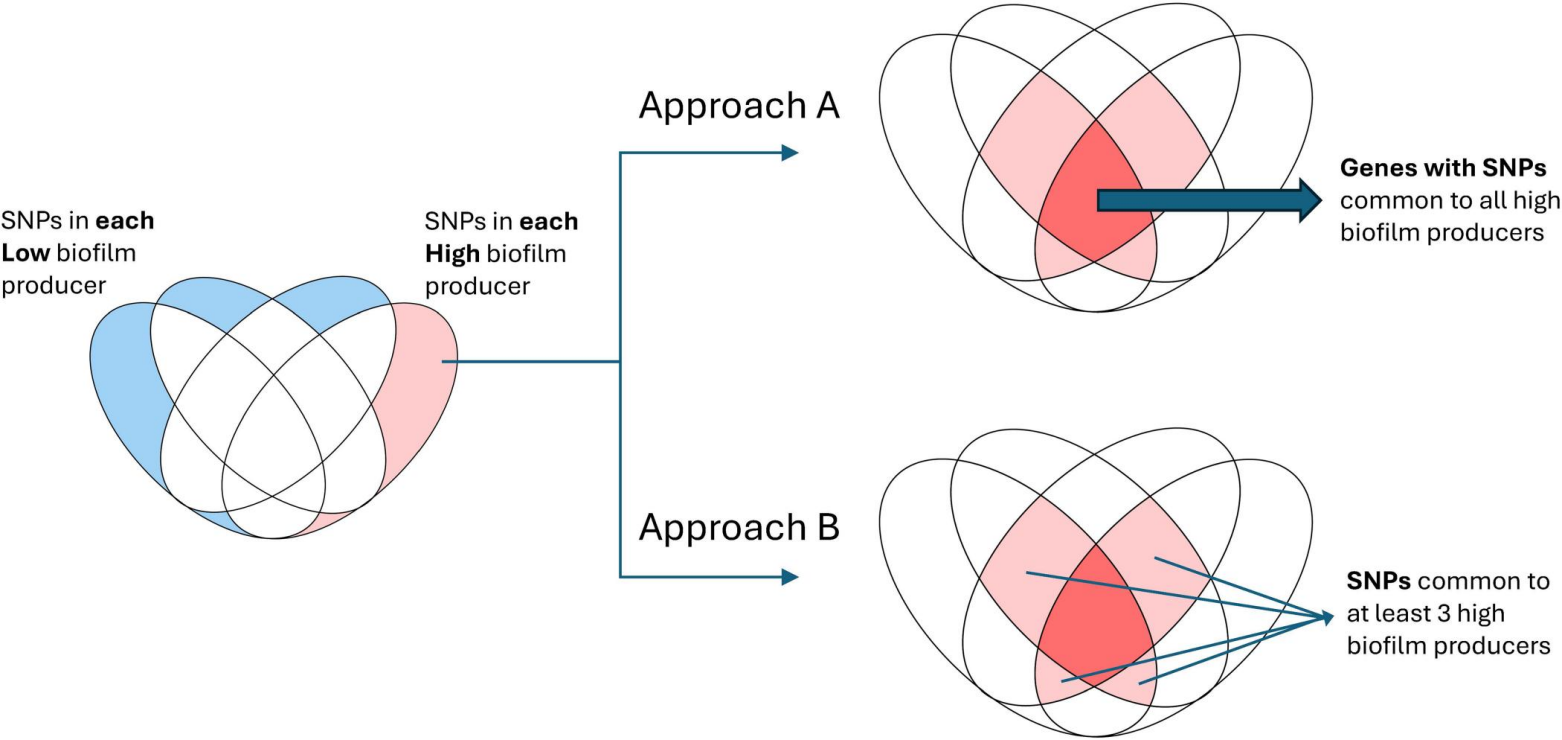
Illustration of the criteria used to identify the most promising biofilm formation‐related genes. As a first step, as illustrated on the left pannel, the SNPs present in each high biofilm producing clinical isolate, and absent in all low biofilm producing clinical isolates, when compared to the reference CBS138 strain, were selected for further analysis. As a second step, as illustrated on the right pannel, one of two approaches were followed: approach A considered all genes with SNPs in common in all the high biofilm producing clinical isolates, yielding the identification of 38 genes of interest; approach B considered all SNPs in common in at least 3 of the high biofilm producing clinical isolates, yielding the identification of 146 genes of interest.

**Table 1:** List of all *N. glabratus* genes, found to have non‐synonymous SNPs present in all the high biofilm producers, but not in the low biofilm producing clinical isolates, when compared to the reference strain CBS138 (Approach A). Genes were organized by their function, orthologs in *S. cerevisiae* and functional descriptions, obtained from the *Candida* Genome Database (www.candidagenome.org).

	*N. glabratus* ORF Name	*N. glabratus* Gene Name	Ortholog in *S. cerevisiae*	Predicted or verified function
Adhesins	*CAGL0J02508g*	*AWP1*		Adhesin‐like protein; belongs to adhesin cluster VI
	*CAGL0G10219g*	*AWP12*		Adhesin‐like protein with 5 tandem repeats; belongs to adhesin cluster VII
	*CAGL0H10626g*	*AWP13*		Predicted cell wall adhesin with a role in adhesion; belongs to adhesin cluster III
	*CAGL0K00110g*	*AWP2*		Putative adhesin; belongs to adhesin cluster V
	*CAGL0A01284g*	*EPA10*		Putative adhesin‐like protein; belongs to adhesin cluster I
	*CAGL0E06666g*	*EPA2*		Epithelial adhesion protein; predicted GPI‐anchor; belongs to adhesin cluster I
	*CAGL0C00110g*	*EPA6*		Sub‐telomerically encoded adhesin; belongs to adhesin cluster I
	*CAGL0I10246g*	*PWP2*		Adhesin‐like protein with tandem repeats; belongs to adhesin cluster II
	*CAGL0I10362g*	*PWP4*		Cell wall adhesin; belongs to adhesin cluster II
	*CAGL0I10098g*	*PWP7*	*FLO9*	Cell wall protein; belongs to adhesin cluster II
	*CAGL0E00231g*			Putative adhesin‐like protein; belongs to adhesin cluster III
	*CAGL0E01661g*			Adhesin‐like protein with tandem repeats; belongs to adhesin cluster III
	*CAGL0H08844g*		*DDR48*	Putative adhesin‐like protein
	*CAGL0J01774g*			Putative adhesin‐like protein; belongs to adhesin cluster VI
	*CAGL0J01800g*			Putative adhesin‐like protein; belongs to adhesin cluster VI
	*CAGL0L00157g*			Putative adhesin‐like protein; belongs to adhesin cluster III
	*CAGL0L00227g*			Putative adhesin; belongs to adhesin cluster V
Genetic regulation	*CAGL0K11396g*	*SIR4*	*SIR4*	Protein involved in subtelomeric silencing and regulation of biofilm formation
	*CAGL0M01716g*	*TEC1*		Transcription factor involved in control of biofilm formation
	*CAGL0F03311g*		*KSP1*	Ortholog(s) have role in protein phosphorylation, regulation of filamentous growth
	*CAGL0A04213g*		*PIN4*	Ortholog(s) have role in DNA damage checkpoint signaling
	*CAGL0M11264g*		*TAF7*	Ortholog(s) have role in RNA polymerase II preinitiation complex assembly
	*CAGL0F06831g*		*MGA2*	Ortholog(s) have role in positive regulation of transcription by RNA polymerase II
	*CAGL0F07381g*			Has role in regulation of DNA‐binding transcription factor activity
Cell wall	*CAGL0J09702g*		*ACK1*	Ortholog(s) have role in fungal‐type cell wall organization
	*CAGL0K12408g*		*BST1*	Ortholog(s) have phosphatidylinositol deacylase activity;
Protein and lipid metabolism	*CAGL0G01430g*		*LAP2*	Ortholog(s) have aminopeptidase activity, role in cellular lipid metabolic process
	*CAGL0L12276g*		*XDJ1*	Ortholog(s) have role in protein import into mitochondrial matrix
	*CAGL0H09130g*		*MNN4*	Ortholog(s) have enzyme activator activity and role in protein N‐linked glycosylation
	*CAGL0J02904g*		*GIP2*	Ortholog(s) have protein phosphatase regulator activity
	*CAGL0J07942g*		*LAM1*	Ortholog(s) have role in intracellular sterol transport
	*CAGL0C01859g*			Has domain(s) with predicted integral component of membrane localization
	*CAGL0C01837g*	*RER1*		Has domain(s) with predicted integral component of membrane localization
Unknown	*CAGL0G07645g*			Protein of unknown function
	*CAGL0L03388g*			Protein of unknown function
	*CAGL0K04873g*		*MFG1*	Protein of unknown function
	*CAGL0K07502g*			Protein of unknown function

Adhesins were the most common assigned category. Several adhesin encoding genes from the *EPA, AWP* and *PWP* families were found with non‐synonymous SNPs in their coding regions, including several putative adhesins, not yet characterized. While the critical role of adhesins in biofilm formation is undeniable, their preponderance in the list of genes with potential biofilm related mutations may result from their genomic locations and repetitive regions, which promote higher probability of stochastic mutations. Interestingly, *TEC1* was found to harbor mutations exclusively in high biofilm‐forming isolates, suggesting a possible contribution to the phenotype. This is consistent with our previous characterization of *TEC1*as a key regulator of biofilm formation in *N. glabratus* (Cavalheiro et al. 2021).

### 3.5 Identification of New Biofilm Formation Effectors Among Genes With Specific SNPs in High Biofilm Producing Strains

Among the 184 genes identified as candidate genes potentially associated with biofilm formation based on SNP enrichment in high‐biofilm producing isolates, 24 were selected for experimental validation because of their higher SNP frequency and because they had no prior association with the biofilm formation phenotype (Table 1 and Table 2). Altogether, regulators of chromatin, transcription, translation and broader cellular organization were selected. The impact of the deletion of each of the selected genes in biofilm formation was assessed through crystal violet assay, using single‐deletion mutants for each of the genes selected and comparing their biofilm formation with that of wild‐type parental strain KUE‐100. This approach was designed to evaluate gene‐level functional relevance rather than the direct effect of the specific clinical‐isolate SNPs. Based on the obtained results, *BST1, EST3, LSM7, AMS1 and ZCF21* were found to be required for maximal biofilm formation in the tested conditions (Figure 7).

**Table 2:** List of selected *N. glabratus* genes, found to have a high frequency of non‐synonymous SNPs present in at least three of the high biofilm producers, but none in the low biofilm producing clinical isolates, when compared to the reference strain CBS138 (Approach B). Genes were organized by their function, orthologs in *S. cerevisiae* and functional descriptions, obtained from the *Candida* Genome Database (www.candidagenome.org).

	*N. glabratus* ORF Name	*N. glabratus* Gene Name	Ortholog in *S. cerevisiae*	Predicted or verified function
Transcription factors	*CAGL0M08800g*	*YAP6*	*CIN5*	bZIP domain‐containing protein
	*CAGL0H01683g*	*ZCF21*	*URC2*	Has domain(s) with predicted DNA‐binding transcription factor activity
	*CAGL0M06831g*	*CRZ1*	*CRZ1*	Transcription factor; downstream component of the calcineurin signaling pathway
	*CAGL0F09097g*	*SKN7*	*SKN7*	Predicted transcription factor, involved in oxidative stress response
	*CAGL0D04136g*		*SRB2*	Has domain(s) with predicted transcription coregulator activity
	*CAGL0E00693g*	*ADA3*	*NGG1*	Putative transcription coactivator, component of the (SAGA) complex
DNA/RNA binding and translation	*CAGL0C03828g*			Best‐hit in *EST3* in *S. cerevisiae* and displays DNA/RNA helicase activity, telomeric DNA binding activity
	*CAGL0L12980g*	*SET1*	*SET1*	Histone methyltransferase, subunit of the COMPASS (Set1C) complex
	*CAGL0M10505g*		*NHP10*	Ortholog(s) have DNA end binding, heteroduplex DNA loop binding activity
	*CAGL0J05764g*		*LSM7*	Ortholog(s) have RNA binding, U2 snRNA binding, poly(U) RNA binding activity
	*CAGL0F01683g*		*RPL22B*	Predicted GPI‐linked protein, ortholog(s) involved in translation
Cyto‐skeleton	*CAGL0B01991g*		*SWF1*	Ortholog has palmitoyltransferase activity and role in cytoskeleton organization
	*CAGL0F03091g*		*DYN2*	Ortholog(s) have plus‐end‐directed microtubule motor activity
Energy	*CAGL0E05148g*		*AMS1*	Ortholog(s) have alpha‐mannosidase activity, role in oligosaccharide catabolic process

**Figure 7:**
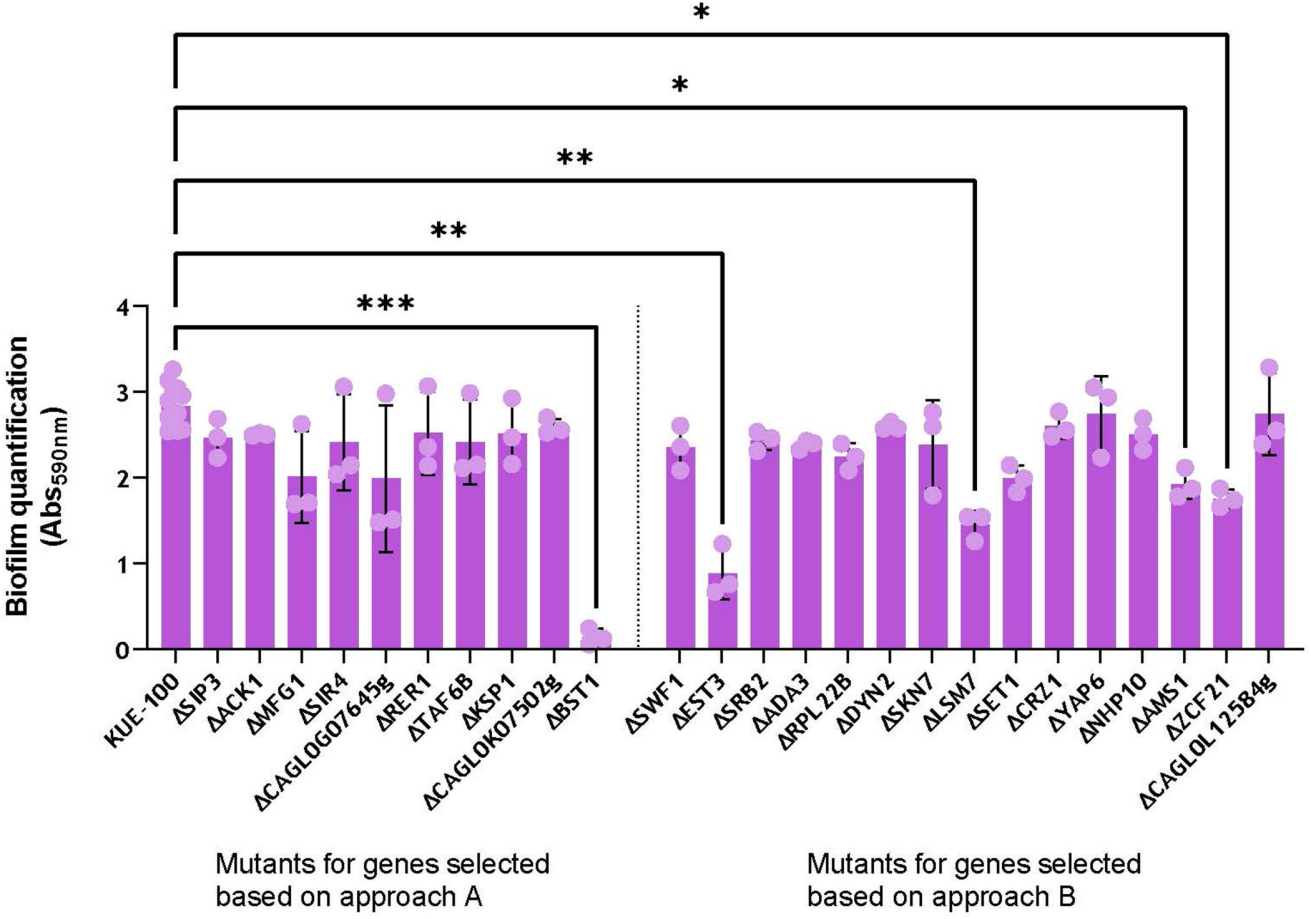
Impact of gene deletion on biofilm formation, assessed by biofilm quantification through the crystal violet assay. Each single deletion mutant is plotted along the horizontal axis, vertical axis plots average value of biofilm formation, through absorbance values at 590 nm. Kruskal‐Wallis nonparametric testing and Dunn's multiple comparison test (correcting for multiple comparisons) was performed, comparing the mean rank of each mutant to the mean rank of the wild‐type strain KUE‐100. Statistically significant differences are highlighted by an asterisk (*), where * = *p* < 0.05, ** = *p* < 0.01 and *** = *p* < 0.001. Average values were calculated from three independent biological sample replicates.

To exclude the possibility that the altered biofilm phenotypes observed in the deletion mutants were primarily caused by defects in cellular growth, growth kinetics of the parental and mutant strains were evaluated through batch cultivation in SDB medium. The growth curves of all the tested strains were virtually indistinguishable, with negligible differences at 24 h, which corresponds to the time point used for biofilm quantification in our experimental conditions (Figure 8).

**Figure 8:**
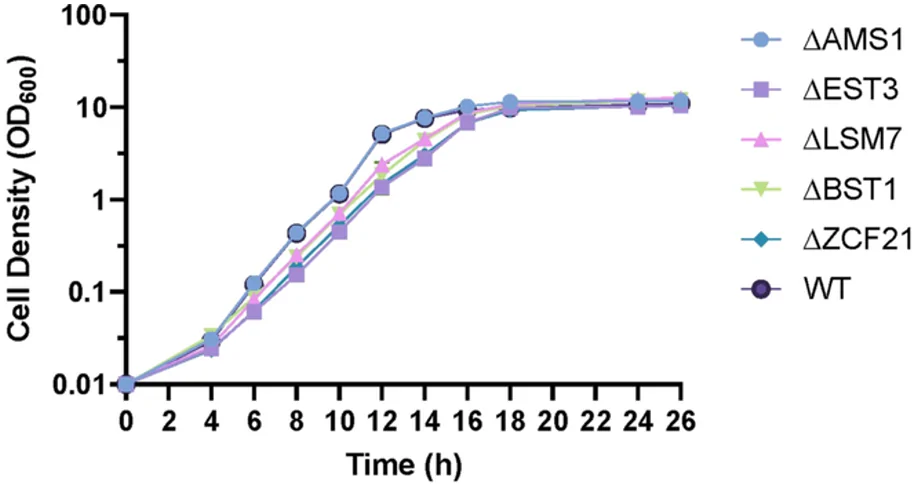
Growth kinetics of *N. glabratus* parental wild‐type (wt) KUE‐100 (purple circles) and derived ∆ams1 (blue circles), ∆est3 (purple squares), ∆lsm7 (pink triangles), ∆bst1 (inverted green triangles), and ∆zcf21 (blue diamonds) single‐deletion mutants was monitored. Growth curves were followed by measuring optical density at 600 nm (OD_600_). Data is presented as mean ± SD from 3 independent biological replicates.

## 4 Discussion

This study focused on extending current knowledge on the phenotypic and genetic diversity underlying biofilm formation in *N*. *glabratus*. The wide variation in biofilm production observed across a collection of nearly 100 clinical isolates is consistent with previous functional genomics studies showing that biofilm formation in *N. glabratus* arises from the contribution of numerous genes and pathways rather than a single dominant factor (Cavalheiro et al. 2021; Maroc et al. 2024; Radchenkova et al. 2025; Beyer et al. 2026; Radchenkova et al. 2025). This complexity supports a model in which different genetic trajectories can converge toward similar biofilm formation phenotypes.

The enrichment of strong biofilm producers among urinary isolates is consistent with the possibility that the urinary tract may impose selective pressures favoring biofilm‐associated traits. Biofilm formation is known to enhance persistence on abiotic surfaces such as catheters and to confer tolerance to antifungal agents, making it a key determinant of chronic infection (Ramage et al. 2001; Andes et al. 2004). These observations align with the idea that *N. glabratus* may adapt to host environments through niche‐specific selection, where traits such as adhesion, matrix production, and stress resistance provide a competitive advantage (Carreté et al. 2019a). One relevant property of the urinary niche is the transit of liquid with greater fluidity, volume and speed compared to the transit of mucus on other anatomic sites (respiratory tract, mouth, vagina). While other anatomical sites harbor well‐characterized microbiota, the urinary tract was long considered sterile, a notion revised by culture‐based studies demonstrating resident microbial communities (Hilt et al. 2014). Additionally, drainage of urine is a common invasive hospital procedure, frequently subjecting this anatomical niche to contact with catheters (Ramage et al. 2001; Andes et al. 2004). Therefore, biofilm production may either be selected by the urinary environment, or simply facilitated by catheterization, as the inert surface of catheters is easily colonized. Either way, the conferred survival advantages ‐ resistance to being swept away by shear forces, resistance to phagocytosis, and resistance to soluble antimicrobial compounds secreted by the uroepithelial cells ‐ promoted by biofilm formation capacity seem mostly convenient for urinary niche colonization (Chandra et al. 2001; Andes et al. 2004).

Interestingly, no correlation between biofilm formation and virulence could be observed. These results suggest that biofilm formation is a complex phenotype that may arise independently of increased virulence. This observation implies that factors other than immune evasion or toxin production—such as host comorbidities and resistance to antifungal treatment—may play a more prominent role in the reported association between *N. glabratus* biofilm formation and adverse outcomes in human disease (Tumbarello et al. 2012; Radchenkova et al. 2025). Moreover, the contribution of biofilm‐forming capacity to infection persistence, which was not assessed in this model, warrants further investigation.

Also, the results obtained suggest that epithelial adhesion and biofilm formation in polystyrene surfaces represent distinct, though potentially complementary, persistence‐associated traits in *N. glabratus*. Increased biofilm‐forming capacity alone does not predict improved adhesion to epithelial surfaces, indicating that adhesion is likely governed by strain‐specific regulatory mechanisms and surface determinants rather than by overall biofilm biomass. As observed in the infection model, this dissociation highlights the complexity of biofilm‐associated phenotypes and supports the notion that biofilm formation may contribute to pathogenicity primarily through persistence, environmental resilience, or treatment tolerance rather than through enhanced initial host cell attachment (Li et al. 2018). Consequently, additional factors—such as differential expression of adhesins, host cell interactions, and environmental cues—are likely to play a critical role in determining colonization efficiency and infection outcome (Timmermans et al. 2018; Cavalheiro et al. 2021).

At the genomic level, the identification of many candidate genes and mutations that may be associated with biofilm formation supports a polygenic architecture for this phenotype. While adhesin‐encoding genes were prominently represented among mutated loci, they were not selected for further analysis due to their localization in subtelomeric regions, which are highly dynamic and subject to rapid mutation and recombination. These regions are known to harbor adhesin gene families such as *EPA* genes, whose expression is tightly regulated by chromatin‐based silencing mechanisms (Rosas‐Hernández et al. 2008; López‐Fuentes et al. 2018). This genomic plasticity, while central to adaptation, complicates the direct association between specific mutations and phenotype. Nonetheless, assessing the impact of the specific SNPs identified in the adhesin encoding genes is worthy of further scrutiny.

In this study, focus was given to 24 candidate genes carrying mutations in high‐biofilm producing clinical isolates but without prior association with biofilm formation in this species. Functional screening through gene deletion in the KUE‐100 background, led to the identification of *BST1*, *EST3*, *LSM7*, *AMS1*, and *ZCF21* as novel contributors to biofilm formation. Notably, these genes are predicted to be involved in diverse cellular processes, including cell wall organization, transcriptional and post‐transcriptional regulation, and genome stability. This functional diversity is consistent with previous findings showing that biofilm formation in *N. glabratus* depends not only on adhesin expression but also on broader regulatory networks controlling gene expression, stress responses, and cellular homeostasis (Cavalheiro et al. 2021). For example, regulators such as Mss11 and Zap1 have been shown to influence biofilm formation by modulating adhesin expression and extracellular matrix composition, illustrating the importance of upstream regulatory control (Gonçalves et al. 2024; Wang et al. 2024).

The identified new effectors of biofilm formation span distinct but potentially interconnected biological processes: *BST1* and *AMS1* are linked to cell wall organization and protein glycosylation, likely affecting adhesion and matrix structure (Liu et al. 2016; Watanabe et al. 2009); *ZCF21* encodes a predicted transcription factor, suggesting a regulatory role(Schillig and Morschhäuser 2013; Mottola et al. 2021); *LSM7* is involved in mRNA processing and turnover, pointing to post‐transcriptional control (Tharun et al. 2000); and *EST3* contributes to telomere maintenance and genome stability (Tuzon et al. 2011), indicating a possible link between cellular fitness and biofilm development. Together, these findings highlight that biofilm formation in *N*. *glabratus* relies on a multilayered network integrating cell surface architecture with gene expression control and cellular homeostasis.

Several of the processes associated with the identified genes are potentially consistent with chromatin‐based regulation mediated by the SIR complex. In *N. glabratus*, biofilm formation is strongly influenced by subtelomeric silencing, which controls the expression of adhesin genes such as *EPA6* through the coordinated action of Sir proteins and chromatin remodeling complexes (Riera et al. 2012). Relief of this silencing by the Swi/Snf complex and other regulators has been shown to enable adhesin expression and biofilm development (Riera et al. 2012). Within this framework, genes such as *ZCF21* and *LSM7* could plausibly modulate transcriptional and post‐transcriptional outputs of SIR‐regulated loci, while *BST1* and *AMS1* may affect the functional deployment of adhesins at the cell surface. Additionally, the involvement of *EST3* raises the possibility of a link between telomere maintenance and subtelomeric gene regulation, as telomere integrity is known to influence chromatin structure and gene silencing at chromosome ends (Park and Lustig 2000; Tuzon et al. 2011).

Overall, this study supports a model in which biofilm formation in *N. glabratus* emerges from the integration of multiple regulatory layers, including chromatin organization, gene expression control, and cell surface architecture. The identification of novel contributors such as *BST1*, *EST3*, *LSM7*, *AMS1*, and *ZCF21* expands the current understanding of this network and highlights the importance of non‐canonical pathways in shaping biofilm phenotypes. These findings further suggest that within‐host evolution, particularly in the urinary tract, may create conditions permissive for the recurrent emergence of genetic variants associated with enhanced biofilm formation through diverse and interconnected mechanisms.

## Author Contributions

**Inês Vieira Costa:** writing – original draft, methodology, validation, formal analysis, data curation, investigation. **Maria Zolotareva:** investigation, methodology, formal analysis, data curation. **Mafalda Cavalheiro:** investigation, methodology. **Dalila Mil‐Homens:** investigation, methodology. **Isabel M. Miranda:** investigation, methodology. **Kaname Sasamoto:** investigation, methodology. **Arsénio Fialho:** supervision, conceptualization. **Acácio G. Rodrigues:** conceptualization, supervision, writing – review and editing. **Hiroji Chibana:** conceptualization, investigation, supervision, writing – review and editing. **Miguel Cacho Teixeira:** conceptualization, funding acquisition, writing – review and editing, supervision, project administration.

## Ethics Statement

The authors have nothing to report.

## Conflicts of Interest

None declared.

## Supporting information


**Table S1:** List of the 95 *N. glabratus* clinical isolates used in this study and associated information on the recovery source (host niche, gender and age), the corresponding Minimum inhibitory concentration (MIC50) values determined for clotrimazole and fluconazole, and biofilm formation, quantification, based on absorbance values at 590 nm (Abs_590nm_), upon the use of the crystal violet assay.

**Table S2:** List of oligonucleotides designed and used in this study for gene disruption, including those used to generate the replacement cassette for each gene and those used for PCR confirmation of gene deletion.

**Table S3:** List of all 146 *N. glabratus* genes found to have a high frequency of non‐synonymous SNPs present in at least three of the high biofilm producers, but none in the low biofilm producing clinical isolates, when compared to the reference strain CBS138 (Approach B). Genes were organized by their function, orthologs in *S. cerevisiae* and functional descriptions, obtained from the Candida Genome Database (www.candidagenome.org).

## Data Availability

The data used to assemble *N. glabratus* genomes of the clinical isolates 21461, 37398, 56067 and 77770 and of the clinical isolates 50570, 50570, 73281 is deposited in NCBI through accession numbers PRJNA1446863 and PRJNA738673, respectively.
